# A functioning Björk–Shiley aortic valve after 36 years without anticoagulation

**DOI:** 10.1093/icvts/ivad029

**Published:** 2023-02-09

**Authors:** Florian Helms, Frauke Rosebrock, Axel Haverich, Saad Rustum

**Affiliations:** Division of Cardiothoracic, Transplantation and Vascular Surgery, Hannover Medical School, Hannover, Germany; Division of Cardiothoracic, Transplantation and Vascular Surgery, Hannover Medical School, Hannover, Germany; Division of Cardiothoracic, Transplantation and Vascular Surgery, Hannover Medical School, Hannover, Germany; Division of Cardiothoracic, Transplantation and Vascular Surgery, Hannover Medical School, Hannover, Germany

**Keywords:** Mechanical valve prosthesis, Björk–Shiley, Anticoagulation

## Abstract

There is a broad consensus among all clinical guidelines that lifelong oral anticoagulation is mandatory after mechanical valve prosthesis implantation. However, in rare cases, patients do not receive anticoagulation or anticoagulation therapy is withdrawn over time. We present a case of an exceptionally durable Björk–Shiley mechanical aortic valve prosthesis still functioning 49 years after implantation. Remarkably, the patient did not receive any anticoagulation or antiplatelet therapy for the first 36 years after implantation. Despite this, no thromboembolic or valve-related adverse events occurred to date. Upon thorough echocardiographic assessment, excellent valve function with a mean transvalvular gradient of 13 mmHg and no prosthetic valve insufficiency was found. This makes the case presented here one of the longest functioning mechanical valve replacements reported.

## INTRODUCTION

The Björk–Shiley (BS) mechanical prosthesis was introduced in 1969 as the first tilting disc valve prosthesis [1]. While current guidelines mandate lifelong anticoagulation after mechanical valve implantation [2], few cases of long-term functioning mechanical valves in patients who did not receive continuous anticoagulation are reported.

## CASE REPORT

**Figure 1:**
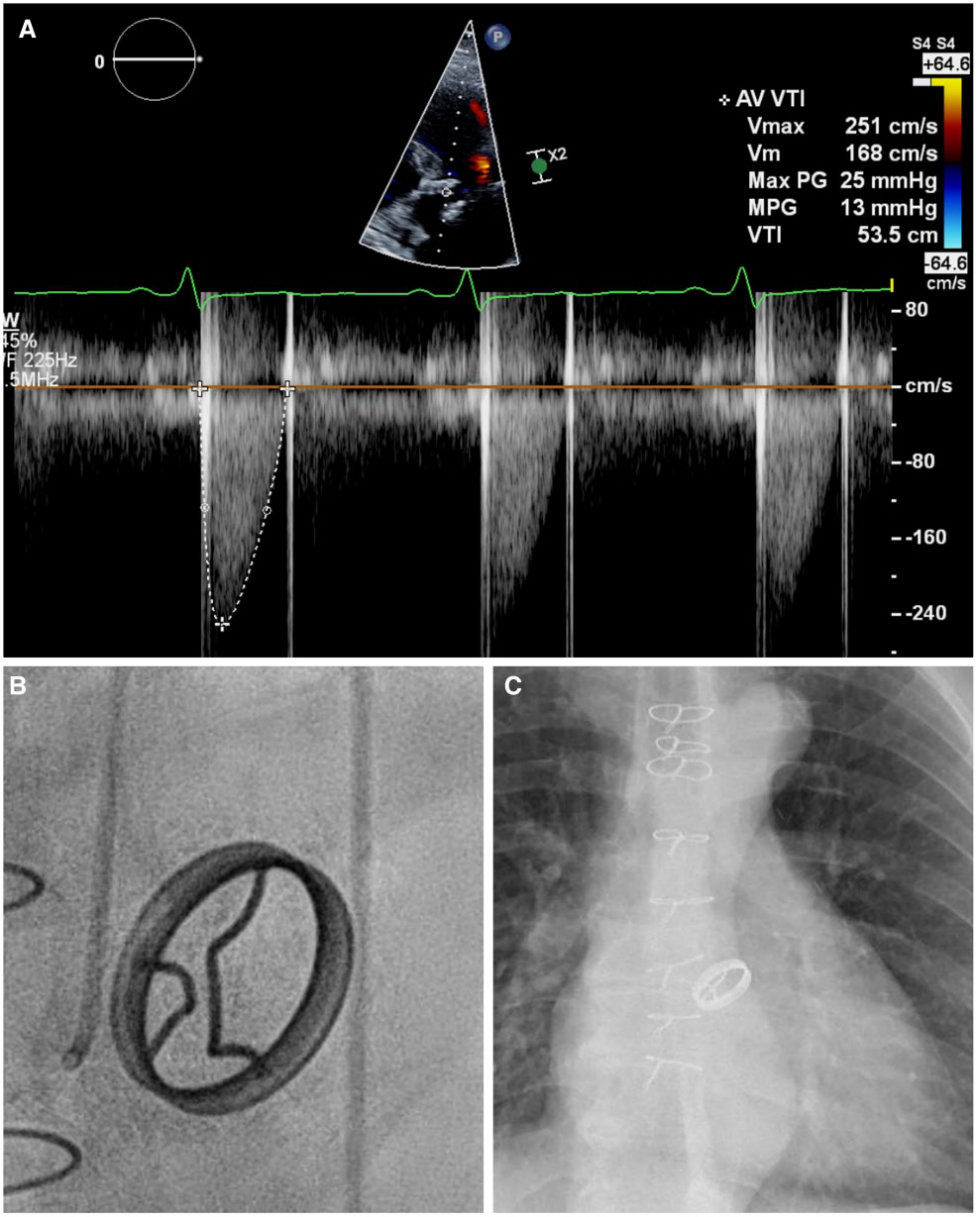
(**A**) Echocardiographic Doppler analysis of the Björk–Shiley valve 49 years after implantation. (**B**) Fluoroscopic aspect during diagnostic coronary angiography. (**C**) X-ray radiographic aspect.

We present a case of a 68-year-old patient who underwent aortic valve replacement with a 25-mm BS Delrin disc prosthesis at the age of 18 years for congenital aortic valve malformation. The patient was initially admitted to our institution with a thoracic aortic aneurysm for replacement of the ascending aorta and aortic arch at the age of 55 years. It was then noted that the patient was not anticoagulated, the international normalized Ratio (INR) was measured at 1.16 and the activated partial thromboplastin time (APTT) was 33 s. The patient reported that he never received oral anticoagulation or antiplatelet medication. Physician’s letters from 1973 confirm that no anticoagulation therapy was prescribed following the initial operation. No altered coagulation status was reported to date. Since the prosthesis was functioning well, no valve replacement was performed. Since the reasons for this extraordinary valve durability in our patient even without proper anticoagulation remain unclear, anticoagulation with phenprocoumon with a target INR of 2.5–3.5 was initiated after the operation. At the time of this report, the patient was readmitted for elective endovascular repair of an abdominal aortic aneurysm. Still, no valve-related adverse events had occurred after 49 years. The patient was asymptomatic and classified in New York Heart Association class I. He was in sinus rhythm and auscultation was normal. In CT angiography, a satisfactory result after the thoracic aortic repair was found. Echocardiography showed normal prosthetic valve function with a maximum and mean transvalvular gradient of 25 and 13 mmHg with a peak velocity of 2.5 m/s (Fig. 1A). No aortic regurgitation was found. Ejection fraction was measured at 60%. Chest X-ray and radiographs acquired during left-heart catheterization showed intact struts and correct valve position (Fig. 1B and C). The operative and postoperative course was uneventful and the patient was discharged home on oral anticoagulation. Informed consent for publication was obtained.

## DISCUSSION

Two further cases of extraordinarily durable aortic BS valves were described (Table 1): Uzun *et al.* [3] reported a 56-year-old patient who underwent BS mechanical valve implantation at the age of 27 for endocarditis. However, a peak gradient of 40 mmHg and grade 1 insufficiency of the valve was found after 29 years. Yildiz *et al.* [4] presented a 42-year-old patient who survived 22 years after BS valve implantation. They report a max/mean gradient of 32/12 mmHg.

**Table 1:** Comparison of reported cases of extraordinarily durable Björk–Shiley valves despite inadequate anticoagulation

	Index patient	Uzun 2001	Yildiz 2006
Age	68	56	42
Ethnicity	White	White	White
Gender	Male	Male	Male
Indication	Congen. heart dis.	Endocarditis	Rheumatism
Size	25	23	n.a.
Years after implantation	49	29	22
Postop anticoagulation duration	None	4 years	None
Years without anticoagulation	36	25	22
Antiplatelet therapy	None	n.a.	None
Auscultation	Normal	Normal	2/6 systolic murmur
TVG peak/mean (mmHg)	25/13	40/n.a.	32/12
Maximum velocity (m/s)	2.5	3.16	n.a.
EF (%)	60	75	n.a.
Insufficiency	None	1st degree	None
INR	1.1^a^	n.a.	0.9
Additional cardiovasc. disease	Aortic aneurysms	Asc. aortic aneurysm	Mitral stenosis

a Measured in 2009.

TVG: transvalvular gradient; EF: ejection fraction; n.a.: no data reported

The durability of the BS valve reported here is remarkable even regardless of the anticoagulation regime. In 2015, Soofi *et al.* [5] claimed to report the ‘Longest in situ working Bjork Shiley valve’ with a 42-year follow-up and continuous anticoagulation. The durability of the BS valve in our patient exceeds this timespan by 7 years. Thus, to the best of our knowledge, we here report the longest functioning BS mechanical aortic valve to date even despite the absence of anticoagulation for the majority of this timespan.

## CONCLUSION

This report is an extreme example of the long-term durability of the first generation BS Delrin valve prosthesis. Nonetheless, lifelong oral anticoagulation must still be considered the gold standard for all mechanical valve prostheses.

**Conflict of interest:** none declared.

## Data Availability

Full data used for this publication is available from the authors upon reasonable request.
